# Atorvastatin, Losartan and Captopril Lead to Upregulation of TGF-β, and Downregulation of IL-6 in Coronary Artery Disease and Hypertension

**DOI:** 10.1371/journal.pone.0168312

**Published:** 2016-12-29

**Authors:** Zahra Sepehri, Mohammad Masoumi, Nazanin Ebrahimi, Zohre Kiani, Ali Akbar Nasiri, Farhad Kohan, Mahmood Sheikh Fathollahi, Mohammad Kazemi Arababadi, Gholamreza Asadikaram

**Affiliations:** 1 Department of Internal Medicine, Faculty of Medicine, Zabol University of Medical Sciences, Zabol, Iran; 2 Cardiovascular Research Center, Institute of Basic and Clinical ‎Physiology Sciences, Kerman University of Medical Sciences, Kerman, Iran; 3 Department of Biochemistry, Faculty of Medicine, Kerman University of Medical Sciences, Kerman, Iran; 4 Student Research Committee, Zabol University of Medical Sciences, Zabol, Iran; 5 Department of Internal Medicine, Faculty of Medicine, Kerman University of Medical Sciences, Kerman, Iran; 6 Department of Anesthesiology, Zabol University of Medical Sciences, Zabol, Iran; 7 Immunology of Infectious Diseases Research Center, Rafsanjan University of Medical Sciences, Rafsanjan, Iran; 8 Department of Social Medicine, Faculty of Medicine, Rafsanjan University of Medical Sciences, Rafsanjan, Iran; 9 Department of Laboratory Sciences, Faculty of Paramedicine, Rafsanjan University of Medical Sciences, Rafsanjan, Iran; 10 Endocrinology and Metabolism Research Center, Institute of Basic and Clinical Physiology Sciences, Kerman University of Medical Sciences, Kerman, Iran; Universidad de Buenos Aires, ARGENTINA

## Abstract

**Introduction:**

Coronary artery disease (CAD) and hypertension are the main reasons of ischemic heart diseases (IHDs). Cytokines as the small glycoproteins are the main arm of immune system and manipulate all of the cardiovascular diseases. The aim of the current study was to examine the effects of treatment of hypertension and CAD on serum levels of IL-6, IL-8, TGF-β and TNF-α.

**Material and Methods:**

This interventional study was performed on the patients with hypertension without CAD (group 1), hypertension and CAD (group 2), CAD but not hypertension (group 3) and without hypertension and CAD as controls (group 4). The patients received routine treatment for hypertension and CAD. Serum levels of IL-6, IL-8, TGF-β and TNF-α were analyzed in the groups treated with various drugs, using ELISA technique.

**Results:**

With regard to the medications, Atorvastatin, Losartan and Captopril were administered more in patients (groups 1, 2 and 3) than the patients without hypertension and CAD. The results revealed that serum levels of TGF-β and IL-6 were significantly increased and decreased, respectively, in the groups 1, 2 and 3 when compared to group 4. Serum levels of TGF-β were also increased in females in comparison to males in the group 4.

**Discussion:**

According to the results it seems that Atorvastatin, Losartan and Captopril have reduced inflammation in *in vivo* conditions via downregulation of IL-6 and upregulation of TGF-β.

## Introduction

Cytokines which are mostly produced by immune cells, are the main arm of immune system and play several roles from inflammation to tissue damage or regeneration [1]. It has been demonstrated that IL-6, IL-8 and tumor necrosis factor-alpha (TNF-α) are the main pro-inflammatory cytokines that are produced by several cells such as macrophages and denderitic cell [2]. The cytokines induce immune cells activation and recruitment and also are the main inducers of arthrosclerosis [3]. TNF-α is the main inducer of infectious shock and can induce hypotension which is the main cause of coma and death in the infected patients [4]. Additionally, tumor growth factor-betta (TGF-β) is an anti-inflammatory cytokine which participates in tissue remodeling and angiogenesis [5]. Based on the fact that during coronary artery disease (CAD) and atherosclerosis the blood tissue is damaged by several agents, like immune cells, it could be hypothesized that the pro-inflammatory cytokines like IL-6, IL-8 and TNF-α and anti-inflammatory cytokines like TGF-β may play key roles in the pathogenesis of the disease [6]. Furthermore, it has been demonstrated that hypertension is a pathologic condition of the cardiovascular system [7]. It is well known that cytokines can manipulate cardiovascular system, like the roles played by TNF-α in the infectious shock [8], it has been hypothesized that cytokines may participate in the induction of hypertension. Previous investigations also confirm that serum levels of IL-6, IL-8, TGF-β and TNF-α were altered in the patients suffering from hypertension and CAD [9, 10]. Aspirin, Clopidogrel, Atorvastatin, Betablokers (Metoprolol and Carvedilol), Nitrates, Losartan and Captopril are the main drug components that are used for treatment of hypertension and CAD [11–13]. The drug components also have anti-inflammatory effects and accordingly, it has been postulated that the drug may improve the hypertension and CAD complications via downregulation of pro-inflammatory molecules. Due to the introduction regarding the roles of cytokines, the main aim of this study was to evaluate the serum levels of IL-6, IL-8, TGF-β and TNF-α in the patients with hypertension and CAD in comparison to individuals without hypertension and CAD who were under treatment with Aspirin, Clopidogrel, Atorvastatin, Metoprolol, Nitrates, Losartan, Captopril and Carvedilol.

## Materials and Methods

### Subjects

This interventional study was performed on a population of Iranian patients with suspected CAD who were candidates for coronary angiography in SHAFA HOSPITAL of Kerman University of Medical Sciences, Iran. Participants filled out a standard questionnaire including demographic information such as age, gender, family history of CAD, education, smoking and use of specific drugs [14]. The inclusion criteria were I) Existence of the ischemic heart disease symptoms, II) Identifying the presence or absence of coronary angiography and III) Acceptance to collaborate in the project. The exclusion criteria were considered as in the previous studies including impossible to investigate the patient exactly and also a history of heart surgery, smoking, congenital heart disease, immune related sickness, nephropathic diseases, and current infectious, diabetes and respiratory diseases, and also physiological conditions related to immune responses such as pregnancy [15]. Given that, all the patients had CAD criteria including chest pain and so on, hence, they were under treatment with Aspirin, Clopidogrel, Atorvastatin, Metoprolol, Nitrates, Losartan, Captopril and Carvedilol. It is worthy to note that all patients with suspected coronary artery disease (CAD) who have been referred for coronary angiography were under treatment for CAD before of coronary angiography, because they were symptomatic, had history of CCU admission or there was evidence of myocardial ischemia in noninvasive study such as exercise test or perfusion imaging. After coronary angiography performed drug administration continued in patients with CAD according to severity of disease and discontinued in patients with normal coronary angiography. The protocol of the study was approved by the Ethical Committee of Kerman University of Medical Sciences.

Selective coronary angiography was performed for all patients according to standard techniques [16]. CAD is defined as luminal diameter narrowing of 50% or more in the main coronary artery vessels, those without any plaque were defined as normal (control group). Hypertension was defined as SBP>140mmHg and/or DBP>90mmHg, and/or on antihypertensive treatment. According to the angiographic data, history, cardiac enzyme assay and electrocardiogram (ECG), participants were divided into four groups as follows: group 1 included 60 patients with hypertension but not CAD, group 2 included 95 patients with hypertension and CAD, group 3 included 61 patients with CAD but not hypertension and group 4 included 70 cases as controls (without hypertension and CAD). The sex, age and smoking matched participants without hypertension and CAD were considered as controls.

5ml blood samples were taken without anti-coagulant pips and centrifuged at 3000 rpm for 10 minutes to separate serum and then kept at -20°C for future cytokine assays.

### Cytokine assay

Serum levels of IL-6, IL-8, TGF-β and TNF-α were evaluated using commercial kits from eBiosciences Company, USA, according to the manufacture's protocols.

### Statistical analysis

Numerical variables are presented as mean ± SE (standard error of mean), while categorical variables are summarized by numbers and percentages.

Serum levels of cytokines were compared using one-way ANOVA followed by Tukey's multiple comparisons test, across the four studied groups. Correlations between BMI and serum levels of the cytokines were evaluated using Spearman' rho correlation of coefficient (r_s_). Also, comparison of drug usage frequencies across the four studied groups was performed using *chi*-square test. Association between quantitative variables was assessed using *Pearson’s* correlation coefficient (r).

For the statistical analysis, the statistical software SPSS version 13.0 for windows (SPSS Inc., Chicago, IL) was used. All p values were 2-tailed, with statistical significance defined by p ≤ 0.05.

## Results

With regard to oral medications before coronary angiography, although administration of Aspirin 80mg daily, Clopidogrel 75mg daily, Betablockers and Nitrates was similar between patients (groups 1, 2 and 3) and control (group 4), Losartan, Captopril and Atorvastatin were administered more to patients in comparison to control group, and these differences were significant (Table 1). Doses of Losartan and Captopril were 50mg twice daily (groups 1 and 2) and 25mg daily (groups 3 and 4). Dose of Atorvastatine was 40mg daily (groups 1, 2 and 3) and 10–20mg for group 4. Accordingly, the control group received significantly lower Atorvastatin (*P* = 0.006), Losartan (*P*< 0.001) and Captopril (*P* = 0.001) than group 1, 2 and 3. Use of other drugs including Aspirin (*P* = 0.541), Clopidogrel (*P* = 0.072), Metoprolol (*P* = 0.304), Nitrates (*P* = 0.567) and Carvedilol (*P* = 0.753) was similar in all groups (Table 1).

**Table 1:** Comparison of drug usage frequencies across the four studied groups.

Drugs	HTN no CAD (n = 60)	HTN Plus CAD (n = 95)	CAD no HTN (n = 61)	Controls (n = 70)	P-value
Aspirin	50 (83.3)	79 (83.2)	55 (90.2)	57 (81.4)	0.541
Clopidogrel	12 (20.0)	37 (38.9)	21 (34.4)	19 (27.1)	0.072
Atorvastatin	42 (70.0)	68 (71.6)	54 (88.5)	43 (61.4)	0.006
Metoprolol	40 (66.7)	49 (51.6)	37 (60.7)	41 (58.6)	0.304
Nitrocantin	38 (63.3)	60 (63.2)	43 (70.5)	41 (58.6)	0.567
Losartan	30 (50.0)	45 (47.4)	2 (3.3)	2 (2.9)	< 0.001
Captopril	8 (13.3)	20 (21.1)	4 (6.6)	1 (1.4)	0.001
Carvediol	2 (3.3)	6 (6.3)	2 (3.3)	2 (2.9)	0.753

Data are presented as n (%).

Frequency of drug usage was compared using *chi*-square test or *Fisher's* exact test across the four studied groups. P < 0.05 was considered as statistically significant difference. HTN: Hypertension, CAD: Coronary Artery Disease.

**Fig 1:**
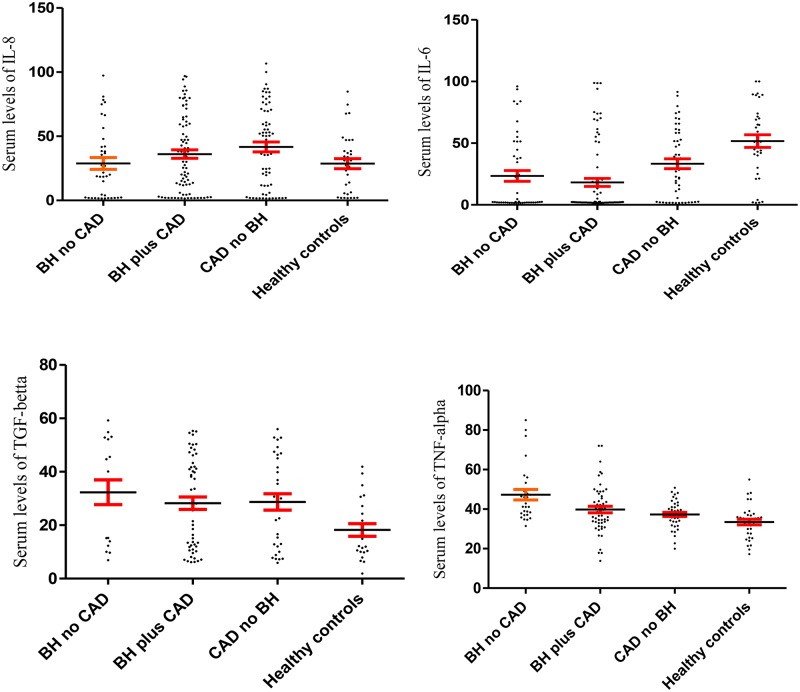
Serum levels of IL-8, IL-6, TGF-β and TNF-α in the four groups including: patients with blood hypertension but not acute coronary syndrome (BH no ACS), patients with blood hypertension and acute coronary syndrome (BH plus ACS), patients with acute coronary syndrome but not blood hypertension (ACS no BH) and healthy controls (without blood hypertension and acute coronary syndrome). The fig shows that the serum levels of IL-6 were significantly decreased and TGF-β were significantly increased in the three patient groups in comparison to healthy controls. Data are displayed as mean (pg/mL) ± SE (standard error of mean).

As mentioned in the Fig 1, the results demonstrated that the serum levels of IL-6 were significantly decreased in group 1 (27.93 ± 5.57), 2 (24.01 ± 4.23) and 3 (39.27 ± 4.41) when compared to control group (59.90 ± 5.64) (p< 0.001). Serum levels of TGF-β in the groups 1, 2, 3 and 4 were 33.37 ± 4.96, 29.70 ± 2.47, 30.48 ± 5.28 and 19.15 ± 1.96 (Fig 1). Statistical analysis demonstrated that serum levels of TGF-β were significantly increased in the patient groups in comparison to control group (group 4) (p = 0.041).

The serum levels of IL-8 (p = 0.920) and TNF-α (p = 0.086) were not statistically different among groups (Fig 1). Data are displayed as mean (pg/mL) ± SE.

The results also showed that serum levels of TGF-β (r_s_ = 0.210, p = 0.012) and TNF-α (r_s_ = 0.153, p = 0.048) have poor correlations with BMI, but IL-6 (r_s_ = 0.075, p = 0.236) and IL-8 (r_s_ = -0.064, p = 0.322) have no correlation with BMI.

**Fig 2:**
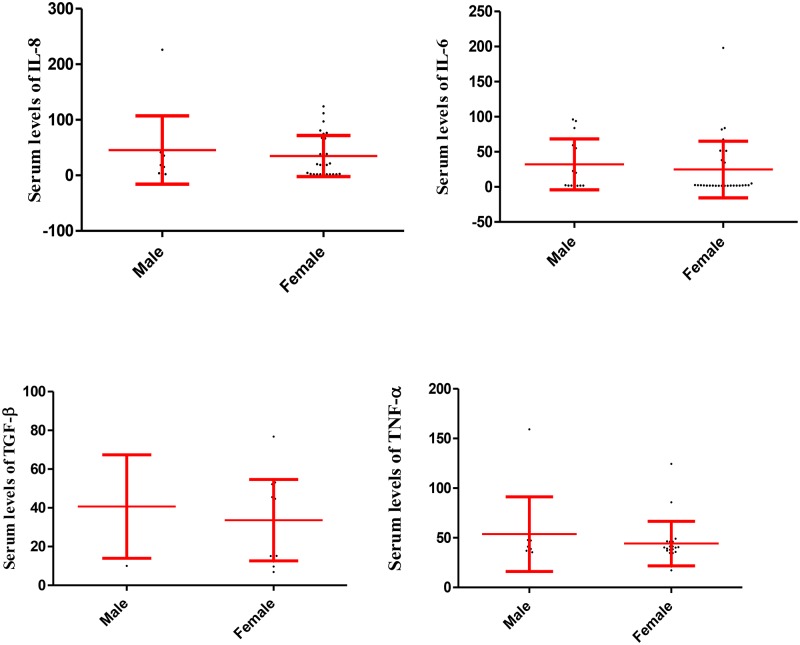
Serum levels of IL-6, IL-8, TGF-β and TNF-α in male and female participants in the group 1. The fig illustrates that serum levels of the cytokines were not significantly different among males and females in the group 1. Data are displayed as mean (pg/mL) ± SE (standard error of mean).

**Fig 3:**
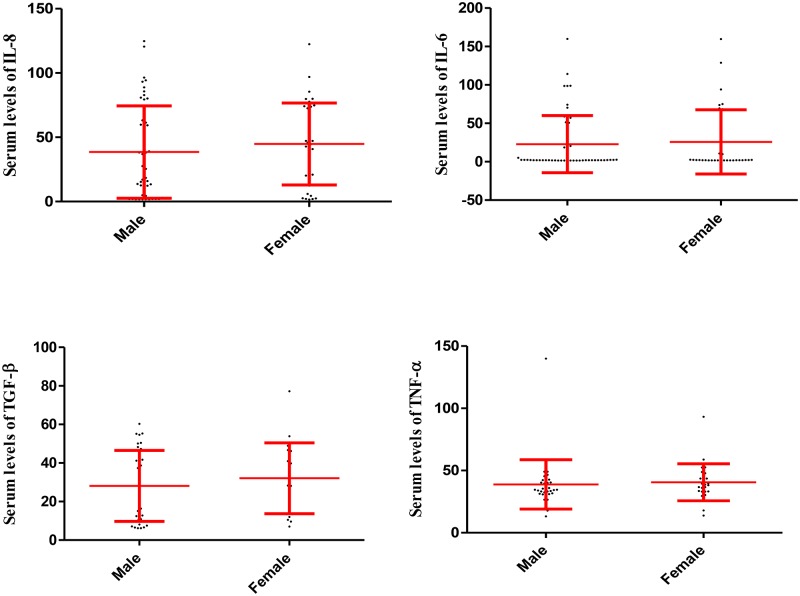
Serum levels of IL-6, IL-8, TGF-β and TNF-α in male and female participants in the group 2. The fig illustrates that serum levels of the cytokines were not significantly different among males and females in the group 2. Data are displayed as mean (pg/mL) ± SE (standard error of mean).

**Fig 4:**
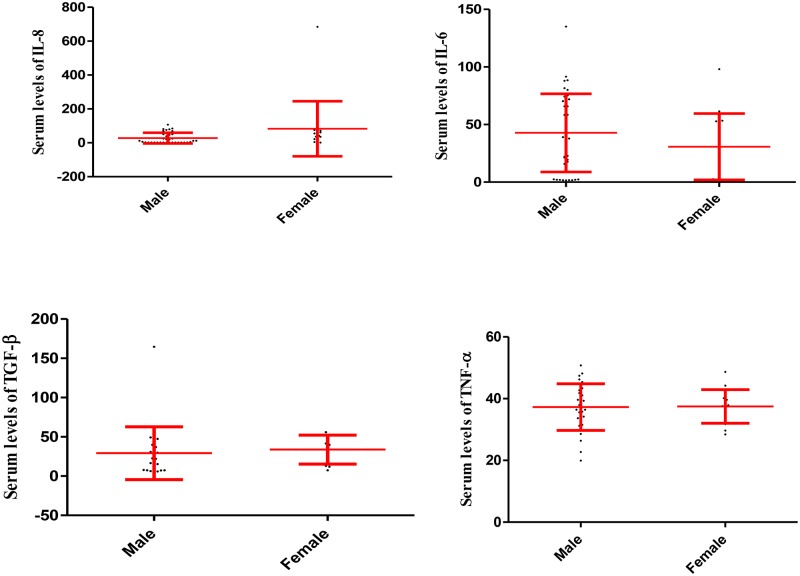
Serum levels of IL-6, IL-8, TGF-β and TNF-α in male and female participants in the group 3. The fig illustrates that serum levels of the cytokines were not significantly different among males and females in the group 3. Data are displayed as mean (pg/mL) ± SE (standard error of mean).

Statistical analysis also revealed that the serum levels of IL-6, IL-8, TGF-β and TNF-α did not differ statistically between males and females in the participants in group 1, 2 and 3, respectively (Figs 2, 3 and 4).

**Fig 5:**
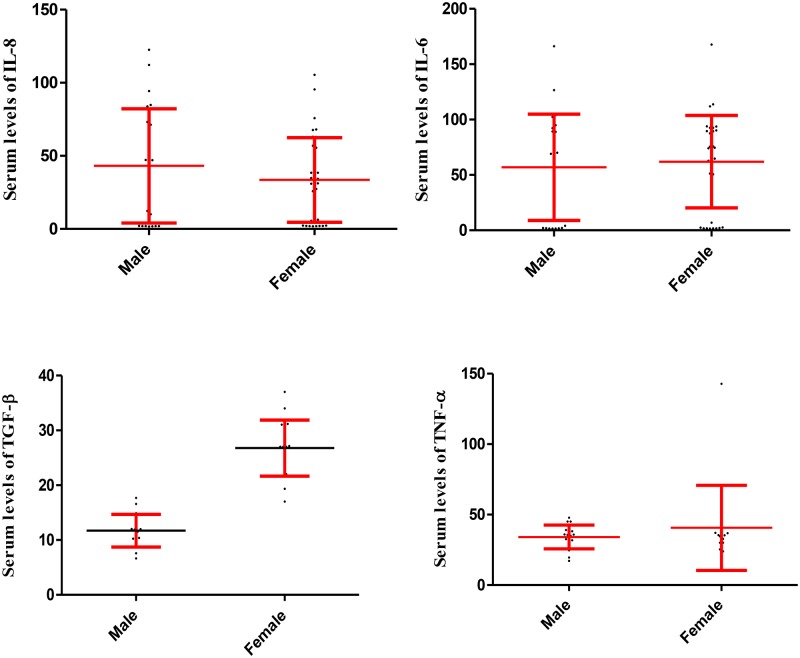
Serum levels of IL-6, IL-8, TGF-β and TNF-α in male and female participants in the group 4. The fig illustrates that serum levels of TGF-β were significantly increased in the females in comparison to males in the healthy controls (group 4). Serum levels of IL-6, IL-8 and TNF-α were not significantly different among males and females in the group 4. Data are displayed as mean (pg/mL) ± SE (standard error of mean).

While serum levels of TGF-β in females were higher than in males (p = 0.003) in the control (Fig 5).

Statistical analysis revealed that, although weight was similar among groups, age was significantly different among groups (Table 2). Post hoc tukey test revealed that only the differences between group 4 vs 2 (p = 0.004) were significant. Additionally, BMI was also different between groups and Post hoc tukey test revealed that only the differences between group 1 vs 2 (p = 0.016) and 1 vs 3 (p = 0.008) were significant (Table 2).

**Table 2:** Demographic information of participants.

Variation	Groups	Mean ± SE	P value
Age	Group 1	54.50 ± 1.21	0.003^1^
	Group 2	58.27 ± 0.97	
	Group 3	57.23 ± 1.23	
	Group 4	52.93 ± 1.33	
Weight	Group 1	70.58 ± 1.95	0.607
	Group 2	68.57 ± 1.46	
	Group 3	67.13 ± 1.85	
	Group 4	67.97 ± 1.77	
BMI	Group 1	26.95 ± 0.68	0.007^2^
	Group 2	24.62 ± 0.46	
	Group 3	24.18 ± 0.54	
	Group 4	25.17 ± 0.56	

The table illustrates that age and BMI were significantly different between groups

^1^ Post hoc tukey test revealed that only the differences between group 4 vs 2 (p = 0.004) were significant.

^2^ Post hoc tukey test revealed that only the differences between group 1 vs 2 (p = 0.016) and 1 vs 3 (p = 0.008) were significant.

Group 1: patients with hypertension without CAD, Group 2: hypertension and CAD, Group 3: CAD but not hypertension, Group 4: without hypertension and CAD as controls (group 4).

Table 3 illustrates that there are positive poor correlations between IL-8 vs TNF-α (.323) and IL-6 vs TGF-β (.343) and also a poor negative correlation between TNF-α vs TGF-β (-.289) in the group 1. There is a good correlation between IL-6 and TNF-α (.612). The correlation between age, weight and BMI with cytokines was too poor to affect their expression in the group.

**Table 3:** Correlations among age, weight, BMI, IL-6, IL-8, TNF-α and TGF-β within group 1 (patients with hypertension without CAD).

		**Age**	**Weight**	**BMI**	**IL-6**	**IL-8**	**TNF-α**	**TGF-β**
**Age**	Spearman Correlation	1.000	-.285	-.180	-0.034	0.174	.038	-0.67
	P value	-	0.027	0.169	0.819	0.270	0.840	0.785
**Weight**	Spearman Correlation	-.285	1.000	.781	.090	.036	.111	-.160
	P value	0.027	-	0.000	0.539	0.821	0.553	0.512
**BMI**	Spearman Correlation	-.180	.781	1.000	.001	-.032	.147	.089
	P value	0.169	0.000	-	0.995	0.843	0.430	0.717
**IL-6**	Spearman Correlation	-0.034	.090	.001	1.000	.158	.612	.343
	P value	0.819	0.539	0.995	-	0.319	0.000	0.151
**IL-8**	Spearman Correlation	.174	.036	-.032	.158	1.000	.323	-.067
	P value	0.270	0.821	0.843	0.319	-	0.093	0.806
**TNF-α**	Spearman Correlation	.038	.111	.147	.612	0.093	1.000	-.289
	P value	0.840	0.553	0.430	0.000	1.000	-	0.362
**TGF-β**	Spearman Correlation	-0.67	-.160	.089	.343	-.067	-.289	1.000
	P value	0.785	0.512	0.717	0.151	0.806	0.362	-

The table illustrates that there are positive poor correlations between IL-8 vs TNF-α (.323) and IL-6 vs TGF-β (.343) and also a poor negative correlation between TNF-α vs TGF-β (-.289). There is a good correlation between IL-6 and TNF-α (.612). The correlation between age, weight and BMI with cytokines was too poor to affect their expression.

Results shows that there is a poor positive correlation between weight vs TGF-β (.284) in group 2 but other correlations were too poor with cytokines to affect their expression (Table 4).

**Table 4:** Correlations among age, weight, BMI, IL-6, IL-8, TNF-α and TGF-β within group 2 (patients with hypertension and CAD).

		**Age**	**Weight**	**BMI**	**IL-6**	**IL-8**	**TNF-α**	**TGF-β**
**Age**	Spearman Correlation	1.000	-.072	-.068	0.047	-.007	.060	-.161
	P value	-	0.489	0.512	0.671	0.951	0.649	0.240
**Weight**	Spearman Correlation	-.072	1.000	.832	.020	-.189	-.040	.248
	P value	0.489	-	0.000	0.860	0.084	0.764	0.67
**BMI**	Spearman Correlation	-.068	.832	1.000	.085	-.195	.008	.199
	P value	0.512	0.000	-	0.440	0.074	0.952	0.145
**IL-6**	Spearman Correlation	.047	.020	.085	1.000	.138	.037	.191
	P value	0.671	0.860	0.440	-	0.213	0.780	0.167
**IL-8**	Spearman Correlation	-.007	-.189	-.195	.138	1.000	.010	.121
	P value	0.951	0.084	0.074	0.213	-	0.939	0.380
**TNF-α**	Spearman Correlation	.060	-.040	.008	.037	.010	1.000	-.009
	P value	0.649	0.764	0.952	0.780	0.939	-	0.959
**TGF-β**	Spearman Correlation	-.161	.248	.199	.191	.121	-.009	1.000
	P value	0.240	0.67	0.145	0.167	0.380	0.959	-

Table illustrates that there is a poor positive correlation between weight vs TGF-β (.284). Other correlations with cytokines were too poor to affect their expression.

In the group 3, there are poor positive correlations between age vs TNF-α (.268), BMI vs IL-8 (.226) and BMI vs TGF-β (.219). There is also a poor negative correlation between BMI and TNF-α (-.252). Other correlations with cytokines were too poor to affect their expression (Table 5).

**Table 5:** Correlations among age, weight, BMI, IL-6, IL-8, TNF-α and TGF-β within group 3 (CAD patients without hypertension).

		**Age**	**Weight**	**BMI**	**IL-6**	**IL-8**	**TNF-α**	**TGF-β**
**Age**	Spearman Correlation	1.000	-.397	-.257	-.095	-.320	.268	-.025
	P value	-	0.002	0.045	0.490	0.020	0.090	0.890
**Weight**	Spearman Correlation	-.397	1.000	.782	.074	.101	-.167	.176
	P value	0.002	-	0.000	0.592	0.473	0.296	0.334
**BMI**	Spearman Correlation	-.257	.782	1.000	.020	.226	-.252	.219
	P value	0.045	0.000	-	0.882	0.103	0.111	0.229
**IL-6**	Spearman Correlation	-.095	.074	.020	1.000	.080	.185	.091
	P value	0.490	0.592	0.882	-	0.580	0.260	0.631
**IL-8**	Spearman Correlation	-.320	.101	.226	.080	1.000	-.089	.161
	P value	0.020	0.473	0.103	0.580	-	0.569	0.388
**TNF-α**	Spearman Correlation	.268	-.167	-.252	.185	-.089	1.000	.003
	P value	0.090	0.296	0.111	0.260	0.569	-	0.990
**TGF-β**	Spearman Correlation	-.025	.176	.219	.091	.161	.003	1.000
	P value	0.890	0.334	0.229	0.631	0.388	0.990	-

The table illustrates that there are poor positive correlations between age vs TNF-α (.268), BMI vs IL-8 (.226) and BMI vs TGF-β (.219). There is also a poor negative correlation between BMI and TNF-α (-.252). Other correlations with cytokines were too poor to affect their expression.

Statistical analysis in the control group (group 4) also demonstrated that there are poor positive correlations between age vs IL-8 (.201), weight vs TNF-α (.278) and moderate positive correlation between BMI vs TNF-α (.436). There is a negative poor correlation between IL-6 and TGF-β (-.251). Other correlations were too poor with cytokines to affect their expression in the group (Table 6).

**Table 6:** Correlations among age, weight, BMI, IL-6, IL-8, TNF-α and TGF-β within group 4 (Controls).

		**Age**	**Weight**	**BMI**	**IL-6**	**IL-8**	**TNF-α**	**TGF-β**
**Age**	Spearman Correlation	1.000	-.211	-.046	.033	.201	.130	.094
	P value	-	0.079	0.706	0.802	0.123	0.451	0.590
**Weight**	Spearman Correlation	-.211	1.000	.817	.134	-.045	.278	.011
	P value	0.079	-	0.000	0.305	0.732	0.101	0.952
**BMI**	Spearman Correlation	-.046	.817	1.000	.146	-.085	.436	.131
	P value	0.706	0.000	-	0.261	0.518	0.008	0.453
**IL-6**	Spearman Correlation	.033	.134	.146	1.000	-.114	.108	-.251
	P value	0.802	0.305	0.261	-	0.394	0.537	0.152
**IL-8**	Spearman Correlation	.201	-.045	-.085	-.114	1.000	.097	-.001
	P value	0.123	0.732	0.518	0.394	-	0.574	0.993
**TNF-α**	Spearman Correlation	.130	.278	.436	.108	.097	1.000	-.192
	P value	0.451	0.101	0.008	0.537	0.574	-	0.369
**TGF-β**	Spearman Correlation	.094	.011	.131	-.251	-.001	-.192	1.000
	P value	0.590	0.952	0.453	0.152	0.993	0.369	-

The table illustrates that there are poor positive correlations between age vs IL-8 (.201), weight vs TNF-α (.278) and moderate positive correlation between BMI vs TNF-α (.436). There is a negative poor correlation between IL-6 and TGF-β (-.251). Other correlations with cytokines were too poor to affect their expression.

## Discussion

It has been documented that most of the drugs which are used for treatment of hypertension and CAD play key roles as anti-inflammatory agents. Previous investigations also confirmed the anti-inflammatory effects of the components. For example, it has been demonstrated that Atorvastatin modulates the innate immune responses by activation of endothelial [17], macrophage [18], monocyte [19], NK cell [19], and neutrophil function [20]. The cells are the main source of pro-inflammatory cytokines including IL-6, IL-8 and TNF-α [21]. TGF-β is also produced by the cells. Accordingly, Blankier et al. reported that Atorvastatin reduced expression of IL-2 and TNF-α by immune cells [22]. Decreased expression of pro-inflammatory cytokines by immune cells under treatment of other drug components including Aspirin, Clopidogrel, Metoprolol, Nitrates, Losartan, Captopril and Carvedilol have also been reported previously [23–25]. Thus, it has been hypothesized that the drugs may downregulate pro-inflammatory cytokines in the patients suffering from hypertension and CAD. Our results showed that all four groups have received all the mentioned drugs, but the control group has received lower doses of Atorvastatin, Losartan and Captopril when compared to other groups (group 1, 2 and 3). Thus, although, we have not evaluated the cytokines serum levels before and after treatment with Atorvastatin, Losartan and Captopril, but based on the results it may be hypothesized that alterations in the cytokine serum levels in the groups suffering from hypertension and CAD in comparison to controls may be associated with the higher doses of the drugs. Thus, more clinical trial investigations using Atorvastatin, Losartan and Captopril can shed light on the understanding of the main mechanisms played by the drugs. The results also revealed that serum levels of IL-6 and TGF-β were significantly decreased and increased, respectively, in the studied groups when compared to control group (without hypertension and CAD). Based on the results it seems that Atorvastatin, Losartan and Captopril may be considered as future target for investigation of their effects on the reduction of IL-6, as a pro-inflammatory cytokine, and increased TGF-β, as an anti-inflammatory cytokine in the studied groups (group 1, 2 and 3). TGF-β is not only an anti-inflammatory cytokine but also plays a key role in repairing the injured tissues including vessels [5]. The cytokine is an angiogeneic factor which actively participates in the development of vessels [5]. Due to the increased expression of TGF-β in the patients with hypertension and CAD who underwent treatment with Atorvastatin, Losartan and Captopril, it may be concluded that the drug components lead to improvement of hypertension and CAD in the specific drug mechanisms and also indirectly via upregulation of TGF-β, which is needed to be evaluated by more clinical trial investigations. It has also been reported that IL-6 is a potential inducer of addressing molecules on the inflamed vessels [21]. Increased expression of the cytokine in the patients suffering from hypertension and CAD has been reported in the previous investigations [26]. Thus, it seems that IL-6 may be considered as a risk factor for hypertension and CAD. Our results revealed that Atorvastatin, Losartan and Captopril may have an indirect therapeutic manner via downregulation of IL-6. Previous investigations on animal models showed that Captopril reduced expression of IL-6 by inhibition of NF-κB, the main transcription factors which transcript from pro-inflammatory cytokine including IL-6 [27]. Another study revealed that Captopril increased expression of TGF-β in animal models [28]. Atorvastatin also reduces expression of pro-inflammatory cytokine by inhibition of NF-κB [29]. Besides, cytokines play important roles in the cells proliferation and also fibrogenic features disorders like atherosclerosis [30]. Moreover, since the significant relations between nerve/cardiovascular systems with immune system via cytokines network were also reported by investigators [31], based on the results presented in the current study it could be assumed that Atorvastatin, Losartan and Captopril, in addition to the known mechanisms, may improve hypertension and CAD via downregulation of pro-inflammatory cytokine and upregulation of anti-inflammatory cytokines, as indirect pathways, and this hypothesis need to be evaluated with more experimental and clinical trial studies. Interestingly, there is a report by Guimarães et al., which showed that Atorvastatin downregulates TGF-β [9]. It appears that the drugs show various features when they are used alone or in combination with other components. Thus, more clinical trials are needed to improve our knowledge regarding the *in vivo* effects of the drug components on human immune system. Our results also demonstrated that serum levels of TGF-β were significantly increased in females when compared to males in the participants without hypertension and CAD (group 4). While no significant association between evaluated cytokines and gender was observed among other groups. It appears that gender has an effect on the production of TGF-β in the patients without hypertension and CAD.

Results revealed that age, BMI and weight have no or poor correlations with the cytokine production. Thus, although the groups differed regarding age and BMI, it seems that the interfered criteria have not affected expression of evaluated cytokines. The results also demonstrated that there is a moderate correlation between IL-6 and TNF-α in the group 1. It has been proven that IL-6 has an agonistic effect with TNF-α. The cytokines are the main cause of macrophage activator and induction of arthrosclerosis. Thus, it seems that although group 1 patients have not been affected by CAD, they are a potential candidate for induction of vessel’s obstruction.

The strengths of the our study is the fact that this study is conducted on human samples and simultaneous analysis of plasma levels of several important cytokines in different conditions in individuals treated with various ranges of drugs. However, the potential limitations of our results were due to the fact that the study were recruited in one centre, which may limit the external validity of our findings, and also some data of the study was limited by questions and observation.

To the best of our knowledge this is the first study which evaluates the effects of hypertension and CAD treatment on the expression of anti-/pro-inflammatory cytokines.

Regarding to wide spread of hypertension and CAD in the world and various ranges of drugs accessible, it is suggested to perform similar study on the other populations to attain more evidences.

### Conclusions

Our study has shown that, Atorvastatin, Losartan and Captopril have reduced inflammation in *in vivo* conditions via downregulation of IL-6 and upregulation of TGF-β.
