# Correction: Atorvastatin, Losartan and Captopril Lead to Upregulation of TGF-β, and Downregulation of IL-6 in Coronary Artery Disease and Hypertension

**DOI:** 10.1371/journal.pone.0172404

**Published:** 2017-02-13

**Authors:** Zahra Sepehri, Mohammad Masoumi, Nazanin Ebrahimi, Zohre Kiani, Ali Akbar Nasiri, Farhad Kohan, Mahmood Sheikh Fathollahi, Mohammad Kazemi Arababadi, Gholamreza Asadikaram

There is an error in affiliation 8 for author Mahmood Sheikh Fathollahi. Affiliation 8 should be: Department of Epidemiology and Biostatistics, Faculty of Medicine, Rafsanjan University of Medical Sciences, Rafsanjan, Iran.

In the Methods section, there is an error in the last sentence of the second paragraph of the section titled “Statistical analysis.” The correct sentence is: Association between quantitative variables was assessed using *Spearman’s* correlation coefficient (r_s_).
